# Residual Disease and HPV Persistence after Cryotherapy for Cervical Intraepithelial Neoplasia Grade 2/3 in HIV-Positive Women in Kenya

**DOI:** 10.1371/journal.pone.0111037

**Published:** 2014-10-24

**Authors:** Hugo De Vuyst, Nelly R. Mugo, Silvia Franceschi, Kevin McKenzie, Vanessa Tenet, Julia Njoroge, Farzana S. Rana, Samah R. Sakr, Peter J. F. Snijders, Michael H. Chung

**Affiliations:** 1 International Agency for Research on Cancer, Lyon, France; 2 Department of Obstetrics and Gynecology, Kenyatta National Hospital, Nairobi, Kenya; 3 Department of Global Health, University of Washington, Seattle, Washington, United States of America; 4 Aga Khan University Hospital, Nairobi, Kenya; 5 Coptic Hospital, Coptic Hope Center, Nairobi, Kenya; 6 Department of Pathology, Vrije Universiteit Medical Center, Amsterdam, the Netherlands; 7 Department of Medicine, University of Washington, Seattle, Washington, United States of America; 8 Department of Epidemiology, University of Washington, Seattle, Washington, United States of America; Penn State University School of Medicine, United States of America

## Abstract

**Objective:**

To assess residual cervical intraepithelial neoplasia (CIN) 2/3 disease and clearance of high-risk (hr) human papillomavirus (HPV) infections at 6 months after cryotherapy among HIV-positive women.

**Design:**

Follow-up study.

**Methods:**

79 HIV-positive women received cryotherapy for CIN2/3 in Nairobi, Kenya, and underwent conventional cytology 6 months later. Biopsies were performed on high grade cytological lesions and hrHPV was assessed before (cervical cells and biopsy) and after cryotherapy (cells).

**Results:**

At 6 months after cryotherapy CIN2/3 had been eliminated in 61 women (77.2%; 95% Confidence Interval, (CI): 66.4–85.9). 18 women (22.8%) had residual CIN2/3, and all these women had hrHPV at baseline. CD4 count and duration of combination antiretroviral therapy (cART) were not associated with residual CIN2/3. CIN3 instead of CIN2 was the only significant risk factor for residual disease (odds ratio, OR *vs* CIN2 = 4.3; 95% CI: 1.2–15.0) among hrHPV-positive women after adjustment for age and HPV16 infection. Persistence of hrHPV types previously detected in biopsies was found in 77.5% of women and was associated with residual CIN2/3 (OR = 8.1, 95% CI: 0.9–70). The sensitivity, specificity, and negative predictive value of hrHPV test in detecting residual CIN2/3 were 0.94, 0.36, and 0.96 respectively.

**Conclusions:**

Nearly one quarter of HIV-positive women had residual CIN2/3 disease at 6 months after cryotherapy, and the majority had persistent hrHPV. CD4 count and cART use were not associated with residual disease or hrHPV persistence. The value of hrHPV testing in the detection of residual CIN2/3 was hampered by a low specificity.

## Introduction

Women living with HIV are at increased risk for infection with human papillomavirus (HPV), cervical intraepithelial neoplasia grade 2 or 3 (CIN2/3) and invasive cervical cancer [1], [2]. Residual or recurrent disease after CIN2/3 treatment is also more frequent among HIV-positive women [3] than HIV-negative women [4], [5].

Most studies of CIN2/3 in HIV-positive women reported outcomes after excisional treatment, e.g. loop electrosurgical excision procedure (LEEP), or cold knife conization (CKC) [3], [6]–[15]. However, cryotherapy is more feasible and affordable than excisional treatment in low- and middle-income countries [16]. Little information is available on the efficacy of cryotherapy for the treatment of CIN2/3 in HIV-positive women [6] and none on the impact of cryotherapy on HPV persistence, i.e., a strong risk factor for residual/recurrent disease in HIV-negative women [4], [5].

The aim of this study was to assess: 1) the frequency of residual CIN2/3 disease and persistent infection of high-risk (hr) HPV types at 6 months after cryotherapy for CIN2/3, and 2) the performance of hrHPV testing after cryotherapy in the detection of residual disease in HIV-positive women in Kenya.

## Methods

### Participants and study procedures

In 2009, 500 HIV-positive women in Nairobi, Kenya were enrolled in a study that compared cervical cancer screening with conventional cytology, visual inspection with acetic acid (VIA) and HPV testing, as described elsewhere [17]–[19]. Briefly, women who attended the Coptic Hope Center for Infectious Diseases for HIV-related conditions were invited to participate and were eligible if they: 1) were between 18 and 55 years of age; 2) had not undergone cervical screening in the last year; and 3) had never been treated for cervical cancer or pre-cancerous lesions. After obtaining a written informed consent, information on women’s characteristics and the use of combined antiretroviral treatment (cART) as well as blood samples to measure CD4 count were collected. Cervical exfoliated cells (further on referred to as “cells”) were obtained using a Cervex-Brush (Rovers Medical Devices, Oss, The Netherlands) and placed in PreservCyt media (Hologic, Marlborough, MA, USA) for HPV testing. A medical doctor performed a colposcopic examination and took a biopsy from all women, either from the most abnormal area on the cervix identified by the colposcopic examination, or at 12 o’clock if no lesion was visualized. Biopsy tissues were immediately immersed in 10% buffered formalin and transported to the local pathology laboratory, where they were embedded in paraffin. Biopsy tissues and PreservCyt media were stored at ambient temperature and shipped to the Department of Pathology at Vrije University Medical Center (Amsterdam, the Netherlands) for HPV DNA testing.

Women who were diagnosed with CIN2/3 disease by biopsy were offered cryotherapy if the lesion was: 1) <75% of the cervix surface and not larger than the cryoprobe tip; and 2) entirely visible and not extending by more than 2 to 3 mm into the endocervical canal [20]. A follow-up visit was planned at 6 months after cryotherapy. It included the collection of cells for conventional cytology and HPV testing and a blood sample for CD4 count. Women with high-grade squamous intraepithelial lesions (HSIL) at cytology were offered a colposcopic examination including a biopsy of the residual lesion. All cytological slides and biopsies were processed under the supervision of the study pathologist (FSR) at the Aga Khan University (Nairobi), who also read all of the cytological and histological slides. Cytology was reported according to the Bethesda 1991 revised classification [21].

### Ethics Statement

The study protocol was approved by the Ethical Review Committees of the Kenyatta National Hospital, Kenya, the University of Washington, the United States, and the International Agency for Research on Cancer, France. The study conformed to the Helsinki Declaration of 1975, as revised in 2000. Each participant gave their written consent.

### HPV DNA testing

HPV DNA testing was done on pre-cryotherapy cells and tissue biopsies and exclusively on cells for the post-cryotherapy visit. Biopsies were sectioned using a ‘sandwich’ approach, whereby inner sections were used for HPV testing and outer sections for histological examination. Testing methods have been described elsewhere [17], [22]. Briefly, detection of HPV DNA was done by GP5+/6+-PCR followed by enzyme immunoassay detection with a cocktail of oligonucleotide probes [23]. Subsequent HPV typing was performed by reverse-line blot hybridization of PCR products [24]. One pre-cryotherapy sample was negative for beta-globin, but positive for hrHPV DNA, and it was included. HPV types 16, 18, 31, 33, 35, 39, 45, 51, 52, 56, 58, 59, and 68 were considered hr types [25].

### Statistical analysis

Women with either histologically confirmed CIN2 or CIN3 were classified as having residual disease; women with either cytology ≤ low-grade squamous intraepithelial lesion (LSIL) or biopsy ≤ CIN1 were classified as cured. Women’s CD4 counts at baseline and at 6-month follow-up were compared using the Wilcoxon signed-rank test.

Age-adjusted odds ratios (ORs) for the presence of residual disease and the corresponding 95% confidence intervals (CIs) were computed. None of the hrHPV-negative women had residual disease. A multiple logistic regression model including age and significant residual disease risk factors was therefore restricted to hrHPV-positive women. In addition, cytology and histology at baseline were closely correlated and we chose to include only histology. Tests for linear trend of ORs were computed giving increasing scores to each level of the categorized variable and fitting them into the model as continuous variables.

The persistence of HPV16, hrHPV types other than HPV16, and the appearance of new hrHPV types were assessed by comparing hrHPV findings from cells or biopsies collected at the pre-cryotherapy visit with those in cells at the post-cryotherapy visit. Crude ORs for residual disease by hrHPV persistence after cryotherapy were computed. Finally, we evaluated the performance of hrHPV testing at 6 months after cryotherapy (sensitivity, specificity, positive predictive value, PPV, and negative predictive value, NPV) for the detection of residual CIN2/3.

## Results

**Figure 1:**
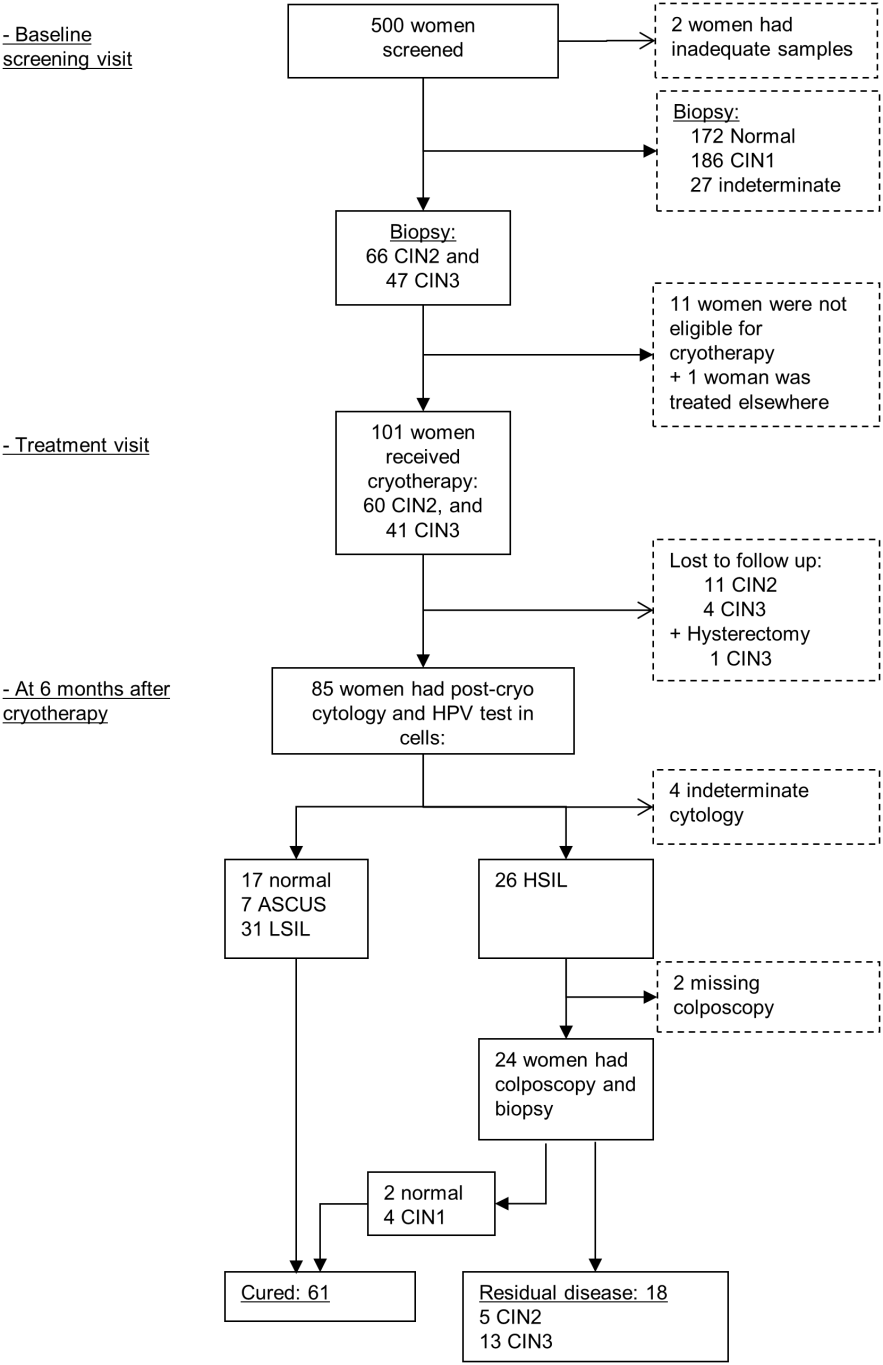
Flowchart study population. HPV, human papillomavirus; CIN, cervical intraepithelial neoplasia; cryo, cryotherapy; ASCUS, atypical squamous cells of undetermined significance; LSIL, low-grade squamous intraepithelial lesion; HSIL, high-grade squamous intraepithelial lesion.

Of the 500 women who were enrolled in our study, 498 had an adequate cell sample (Figure 1). CIN2 and CIN3 were detected in 66 and 47 women, respectively, and 101 received cryotherapy 30 days on average after inclusion in the study. A follow-up visit was scheduled at 6 months after cryotherapy (median: 182 days, Interquartile range, IQR: 165–197). Fifteen women (11 CIN2 and 4 CIN3) did not attend the follow-up visit and one woman with CIN3 underwent hysterectomy. Out of the remaining 85 women, 81 had a valid cytology and 26 had HSIL. All but two women with HSIL underwent colposcopic examination and biopsy collection. Among 18 women with residual disease, 5 had CIN2 and 13 CIN3. Only 17 women had negative cytology at the follow-up visit. Sixty-one women who had either cytology ≤ LSIL or biopsy ≤ CIN1 were considered cured (Figure 1).

The median age of 79 women eventually included in the present report was 41 years (IQR: 35–45). At baseline, 61 (77.2%) of them were using cART, and 6 had started cART between the baseline and follow-up visit. Median CD4 count was lower at baseline (322 cells/µL, IQR: 205–477) than at the follow-up visit (386, IQR: 272–558) (p = 0.007).

Table 1 shows OR for residual CIN2/3 by selected baseline characteristics. Cryotherapy eliminated CIN2/3 in 77.2% (95% CI: 66.4–85.9) of all women (Table 1). The presence of HSIL cytology (OR *vs* ≤ LSIL = 5.2; 95% CI: 1.1–25.0); CIN3 in biopsy (OR *vs* CIN2 = 5.3; 95% CI: 1.6–17.4); infection with hrHPV (OR = ∞; 95% CI: 1.9-∞), and HPV16 (OR = 3.9; 95% CI: 1.1–13.1) in cells at baseline were significantly associated with residual disease in the age-adjusted models. Parity, CD4 cell count, duration of cART use, diagnosis at VIA, and multiple hrHPV infections at baseline (37.7% of hrHPV-positive women) were unrelated to residual disease. Residual disease was only found among 61 women who were hrHPV-positive at baseline and, among them, 70.5% (95% CI: 57.4–81.5%) were cured. The presence of CIN3 instead of CIN2 at baseline was significantly associated with residual disease (OR *vs* CIN2 = 4.3; 95% CI: 1.2–15.0) after adjustment for age and HPV16 infection (Table 1).

**Table 1:** Odds ratio (OR) and 95% confidence intervals (CI) for residual disease six months after cryotherapy (cryo) for CIN2/3 by selected pre-treatment characteristics.^1^Kenya, 79 HIV-positive women.

Pre-cryocharacteristics	Total N	Cured(≤LSIL/CIN1)n (%)	Residualdisease(CIN2/3)n (%)	OR^2^(95% CI)	OR^3^(95% CI)
**Total**	79	61 (77.2)	18 (22.8)		
**Age**					
<35	19	17 (89.5)	2 (10.5)	1	1
35–44	39	28 (71.8)	11 (28.2)	3.3 (0.7–16.9)	3.3 (0.6–19.4)
≥45	21	16 (76.2)	5 (23.8)	2.7 (0.5–15.7)	2.2 (0.3–14.7)
*χ* 2 *_1_ for trend*				*p = 0.340*	*p = 0.543*
**Parity**					
0–1	21	19 (90.5)	2 (9.5)	1	
2	23	18 (78.3)	5 (21.7)	2.2 (0.4–13.6)	
≥3	35	24 (68.6)	11 (31.4)	3.5 (0.6–19.8)	
*χ* 2 *_1_ for trend*				*p = 0.153*	
**CD4 cells/µL at baseline**					
>500	16	12 (75.0)	4 (25.0)	1	
200–500	45	35 (77.8)	10 (22.2)	1.0 (0.3–4.0)	
<200	18	14 (77.8)	4 (22.2)	1.1 (0.2–6.0)	
*χ* 2 *_1_ for trend*				*p = 0.891*	
**CD4 count/µL at 6-month follow-up**					
≥500	22	16 (72.7)	6 (27.3)	1.0	
200–500	47	37 (78.7)	10 (21.3)	0.7 (0.2–2.6)	
<200	10	8 (80.0)	2 (20.0)	0.8 (0.1–5.9)	
χ^2^ *_1_* for trend				*p = 0.750*	
**cART**					
No	18	16 (88.9)	2 (11.1)	1	
<2 years	37	28 (75.7)	9 (24.3)	2.1 (0.4–11.7)	
≥2 years	24	17 (70.8)	7 (29.2)	2.5 (0.4–15.3)	
*χ* 2 *_1_ for trend*				*p = 0.343*	
**VIA**					
Neg	33	28 (84.9)	5 (15.2)	1	
Pos	46	33 (71.7)	13 (28.3)	2.6 (0.8–8.6)	
**Cytology** 4					
Normal/ASCUS/LSIL	25	23 (92.0)	2 (8.0)	1	
HSIL or worse	52	36 (69.2)	16 (30.8)	5.2 (1.1–25.0)	
**Histology**					
CIN2	45	40 (88.9)	5 (11.1)	1	1
CIN3	34	21 (61.8)	13 (38.2)	5.3 (1.6–17.4)	4.3 (1.2–15.0)
**hrHPV (cells)**					
Neg	18	18 (100)	0	1	
Pos	61	43 (70.5)	18 (29.5)	∞ (1.9 - ∞)	
**HPV16 (cells)**					
Neg	64	53 (82.8)	11 (17.2)	1	1
Pos	15	8 (53.3)	7 (46.7)	3.9 (1.1–13.1)	2.2 (0.6–8.1)
**Multiple hrHPV** 5 **(cells)**					
No	38	25 (65.8)	13 (34.2)	1	
Yes	23	18 (78.3)	5 (21.7)	0.6 (0.2–1.9)	

1 Unless otherwise specified;

2 Adjusted for age as appropriate;

3 Among 61 hrHPV-positive women only. Adjusted for age, histology and HPV16 as appropriate;

4 Do not add up to the total because of 2 indeterminate cytology results;

5 Among hrHPV-positive women only.

cART, combination antiretroviral therapy; VIA, visual inspection with acetic acid; hrHPV, high-risk human papillomavirus; ASCUS, atypical squamous cells of undetermined significance; LSIL, low-grade squamous intraepithelial lesion; CIN, cervical intraepithelial neoplasia.

After cryotherapy, hrHPV infection in cells was detected in 56 (70.9%) women, of whom 22 (39.3%) had multiple hrHPV types, and 2 were new infections in women who were previously hrHPV-negative. A comparison of hrHPV findings at baseline and 6 months after cryotherapy among women who were initially hrHPV-positive in cells (n = 61) or biopsies (n = 49) is shown in Table 2. Persistence of at least one hrHPV type in cells was detected in 80.4% of women, including persistence of HPV16 in 14.8%. New hrHPV types, in the lack of persistent types, were detected in 5 women whereas 7 (11.5%) had become hrHPV-negative. Similar proportions of persistence were found when the comparison was based on hrHPV types detected in pre-treatment CIN2/3 biopsies (Table 2). The persistence of HPV16 or other hrHPV types was not influenced by CD4 count or duration of cART use (data not shown).

**Table 2:** Presence of hrHPV after cryotherapy (cryo) by type of pre-cryo sample in women who were hrHPV-positive before cryotherapy.

Post-cryo hrHPV status	Pre-cryo hrHPV-positive women	
Cells	Cells n (%)	Biopsy n (%)
**Persistent, HPV16**	9 (14.8)	6 (12.2)
**Persistent, other hr types** 1	40 (65.6)	32 (65.3)
**New type(s) only** 2	5 (8.2)	6 (12.2)
**Negative**	7 (11.5)	5 (10.2)
**Total**	61	49^3^

Kenya, 61 HIV-positive women.

1 Persistence of at least one hr type but not HPV16.

2 In the absence of persistent hr types.

3 Does not include 12 women who were hrHPV-positive in cells but negative in biopsy.

hrHPV, high-risk human papillomavirus.

Table 3 shows OR for residual disease according to the presence of persistent hrHPV infection after cryotherapy. Women with persistent infection showed an increased risk of residual disease. ORs were 5.8 (95% CI: 0.7–49.2); and 8.1 (95% CI: 0.9–69.7) for persistence of types previously detected in baseline cells or biopsy, respectively. The level of persistence of hrHPV infections was unrelated to the level of CD4 count or the use of cART (data not shown). None of the five women with new hrHPV types in the lack of persistent infection showed residual disease (data not shown).

**Table 3:** Crude odds ratio (OR) and 95% confidence intervals (CI) for residual disease six months after cryotherapy (cryo) for CIN2/3 by persistence of hrHPV infections detected in pre-cryo cells or biopsies.

Persistence ofhrHPV	Total n	Cured (≤LSIL/CIN1) n (%)	Residual disease(CIN2/3) n (%)	OR (95% CI)
**Pre-cryo cells**				
No	12	11 (25.6)	1 (5.6)	1
Yes	49	32 (74.4)	17 (94.4)	5.8 (0.7–49.2)
**Pre-cryo biopsies** 1				
No	11	10 (32.3)	1 (5.6)	1
Yes	38	21 (67.7)	17 (94.4)	8.1 (0.9–69.7)

Kenya, 61 HIV-positive women.

1 Does not include 12 women who were hrHPV-positive in cells but negative in biopsy.

hrHPV, high-risk human papillomavirus; LSIL, low-grade squamous intraepithelial lesion; CIN, cervical intraepithelial neoplasia.

The performance of post-treatment hrHPV testing to detect residual disease is shown in Table 4. The sensitivity and specificity of hrHPV testing at 6 months after cryotherapy were 0.94 and 0.36, respectively; PPV and NPV were 0.30 and 0.96, respectively. Testing for either or both HPV16 and 18 showed lower sensitivity and NPV than hrHPV testing, but better specificity and PPV (except for HPV18 only). Persistent hrHPV infection showed sensitivity, specificity, PPV and NPV of 0.94, 0.48, 0.35 and 0.97, respectively.

**Table 4:** Performance of hrHPV testing for the detection of CIN2/3 six months after cryotherapy.

Post- treatment	TP	FP	FN	TN	Sensitivity	Specificity	PPV	NPV
hrHPV	17	39	1	22	0.94 (0.73–1.00)	0.36 (0.24–0.49)	0.30 (.19–0.44)	0.96 (0.78–1.00)
HPV16	7	6	11	55	0.39 (0.17–0.64)	0.90 (0.80–0.96)	0.54 (0.25–0.81)	0.83 (0.72–0.91)
HPV18	4	8	14	53	0.22 (0.06–0.48)	0.87 (0.76–0.94)	0.33 (0.10–0.65)	0.79 (0.67–0.88)
HPV16/18	11	14	7	47	0.61 (0.36–0.83)	0.77 (0.65–0.87)	0.44 (0.24–0.65)	0.87 (0.75–0.95)
Persistent hrHPV^1^	17	32	1	29	0.94 (0.73–1.00)	0.48 (0.35–0.61)	0.35 (0.22–0.50)	0.97 (0.83–1.00)

Kenya, 79 HIV-positive women.

1 At least 1 same hrHPV type present before and 6 months after cryotherapy.

TP, true positive, FP, false positive, FN, false negative, TN, true negative, PPV, positive predictive value, NPV, negative predictive value, hrHPV, high-risk human papillomavirus.

## Discussion

Our study on the efficacy of cryotherapy for the treatment of CIN2/3 in HIV-positive women is one of the few that included cytological and histological evaluation prior to and after treatment and information on treatment outcome by persistence of individual hrHPV types. Cryotherapy eliminated CIN2/3 in 77% (95% CI: 66–86) of all women and 71% (95% CI: 57–82%) of women who had hrHPV infection before treatment, but only a small minority of them became hrHPV-negative. Contrary to others [8], [11], [13], [26], we chose to define as cure the elimination of CIN2/3 rather than the disappearance of any cytological abnormalities. If we had used normal cytology as threshold, only 19 (24.1%) of our study women would have been considered cured. However, on account of the difficulty to eliminate HPV infection and its morphological correlate (low-grade lesions) in HIV-positive women, we reasoned that the cure of CIN2/3 had to be considered the most important outcome.

An initial diagnosis of CIN3, instead of CIN2, was the most important predictor of residual disease. HPV16-positivity at baseline was not significantly associated with residual disease after adjustment for the presence of CIN3. Infections with multiple hrHPV types were relatively frequent (38%) in study women but they did not increase the probability of residual disease as expected since cancer progression-prone CIN2/3 are assumed to be monoclonal [27], [28]. No residual disease was found among 18 women who were initially hrHPV-negative.

Some [8], [11], [13], [26], but not all studies [3], [9], [12], reported that cART treatment or high CD4 counts were favourable prognostic factors for treatment outcome. In our cross-sectional study, neither cART use nor CD4 counts prior to or after cryotherapy were associated with residual disease. Unfortunately, we did not have systematic information on CD4 count prior to cART. However, cART tended to be started relatively late in Kenya in 2009 (i.e, CD4 count <250 cells/µL or presence of WHO clinical III/IV stage disease) [29], when CIN2/3 may have already become refractory to immune reconstitution [17].

Our study confirms that residual disease after excisional treatment or cryotherapy of CIN2/3 may be more frequent among HIV-positive women than HIV-negative women. A study from Zimbabwe [6] reported residual CIN2/3 at 6 months after cryotherapy or LEEP in 3.8% and 1.8% of 109 HIV-positive women, respectively, but in none of 38 HIV-negative women. A few additional reports mainly from high-income countries included only HIV-positive women and consistently showed the difficulty in achieving cure of CIN2/3 and avoiding long-term recurrences in HIV-positive women. Tebeu et al [30] reviewed early studies on the outcome of conization for any degree of CIN in HIV-positive women at 6-to-74 months after treatment. Recurrences ranged between 20% and 75% but residual CIN1 was not separated from CIN2/3. Heard et al [11] reported that 54% of 75 HIV-positive women treated with LEEP or conization in France had a CIN recurrence at 36 months, and approximately one third had CIN2/3. Massad et al [26] reported residual CIN2/3 disease in 12% of 115 mostly HIV-positive women mainly treated with excisional methods in the United States. Reimers et al [3] detected residual CIN2/3 in 42% of 75 HIV-positive women at 6 months after LEEP or CKC.

Rates of residual disease and recurrences in HIV-negative women after CIN2/3 treatment were reported in many studies [31], [32]. One study of 610 women from the United States showed residual CIN2/3 at 6 months after LEEP in 3% of women, and 7% after 2 years [4]. In other studies in which excisional methods were used for CIN2/3 treatment, residual disease ranged between 1% to 8% at 6 months [33], [34], and between 2% and 20% at 2 years [33], [35], [36] or more [37]. A few studies from India [38], [39] and Africa [6] showed persistent or recurrent diseases in 13% or less of HIV-negative women at 2 years or more after cryotherapy.

The difference in hrHPV clearance between HIV-positive and HIV-negative women after CIN2/3 treatment is much larger than the difference in the frequency of residual disease. Positivity for hrHPV after LEEP was shown in 57% of HIV-positive women with any degree of CIN by Gingelmaier et al [10] in Germany. The proportion of HIV-negative women who were hrHPV-positive at 6 months after treatment in PCR-based studies was 10 to 37% [4], [37], , except in studies that used ultra-sensitive HPV tests [33], [36]. Type-specific hrHPV persistence in our study (80%) was also larger than that reported after cryotherapy or excisional treatment among HIV-negative women (7-to-37%) [4], [40], [43]. The association between persistent hrHPV infection and residual disease in our study was especially strong (8-fold increase, of borderline statistical significance) when the persistent type was the one previously detected in the CIN2/CIN3 biopsy.

A positive association between persistent HPV infection and residual or recurrent CIN2/3 was shown in HIV-negative women [4], [37], [41], [44] lending support to the use of HPV testing to detect treatment failure [31]. In fact, HPV testing was shown to be more sensitive and not significantly less specific than cytology screening in a large meta-analysis that excluded verification bias [31]. The sensitivity of hrHPV testing in our study (94%) was similar to the corresponding sensitivity in HIV-negative women (92%) [31] but specificity (36%) was much lower than among HIV-negative women (76%) [31]. Sensitivity did not change but specificity improved to 48% when we used persistent hrHPV infection instead of hrHPV-positivity after cryotherapy. Testing for HPV16 and/or 18 showed a specificity of 77% but a sensitivity of only 61%. An equally valid evaluation of the performance of cytology testing in HIV-positive women was impossible in our study due to verification bias, as only women with HSIL cytology received colposcopic examination.

Limitations of our study include the lack of random biopsies at follow-up visit and the short follow-up. A 6-month follow-up prevented the evaluation of long-term recurrences after cryotherapy in HIV-positive women and lends support to the interpretation of treatment failures as residual disease. Infections with new hrHPV types were seldom detected. We were also unable to compare the efficacy of cryotherapy to excisional methods as they were used only exceptionally. However, a meta-analysis [32] did not show systematic differences across seven different techniques of CIN treatment, including cryotherapy, in HIV-negative women. Strengths of our study include the use of high-quality cytological and histological examination and a clinically validated HPV testing method [45] on pre- and post-treatment cells and pre-treatment CIN2/3 biopsies.

In conclusion, we demonstrated that cryotherapy eliminated three quarters of CIN2/3, but only one fifth of hrHPV infections in HIV-positive women. HIV-positive women should be, therefore, carefully monitored after cryotherapy as they often remain cytologically abnormal and hrHPV-positive. In fact, the value of testing for hrHPV infection to detect residual CIN2/3 after cryotherapy was hampered by the lower specificity of the test than in HIV-negative women. The clinical implications of low hrHPV clearance will require further study and so will the assessment of the possible influence of long-term history of cART use and CD4 count on recurrence rates.
